# Supplementary material for: Complexome profiling of the Chlamydomonas psb28 mutant reveals TEF5 as an early PSII assembly factor
Source: Plant Cell. 2025 Mar 17;37(6):koaf055. doi: 10.1093/plcell/koaf055 (PMC12164590; doi:10.1093/plcell/koaf055)
Supplement: koaf055_Supplementary_Data [file koaf055_supplementary_data.zip › psb28_interactive.zip!/plots/intensity/qualityPlots/peptides/_Cre13.g604150.t1.2.html]

### Cre13.g604150.t1.2

Trivial name:   
SeqCoverage [median]: 8.80  
Mass: 97.98 kDa  
  
 

| Sequence | MissedCleavage | Mass [Da] | wtIntensity | muIntensity | Score |
| --- | --- | --- | --- | --- | --- |
| EVNALDNATSSK | 0 | 1247.60 | 0.00 | 342.64 | 134.68 |
| HAVAVAAGGK | 0 | 879.49 | 707.55 | 0.00 | 121.29 |
| LHDTVSSLPEGYNTVVGER | 0 | 2072.02 | 0.00 | 2339.76 | 83.25 |
| SALFVAHR | 0 | 899.50 | 0.00 | 445.40 | 102.71 |
| VAPGESVAVVGSSGSGK | 0 | 1486.76 | 451.08 | 688.22 | 106.58 |
| YGRPDATDAEVVAAAVAAR | 1 | 1901.96 | 0.00 | 2802.80 | 71.60 |
|  |  |  | 1158.6 | 6618.8 |  |
